# Associations between ultra-processed foods intake and preserved ratio impaired spirometry in U.S. adults

**DOI:** 10.3389/fnut.2025.1523736

**Published:** 2025-01-31

**Authors:** Weiliang Kong

**Affiliations:** Department of Respiratory and Critical Care Medicine, Key Laboratory of Respiratory Disease of Ningbo, The First Affiliated Hospital of Ningbo University, Ningbo, China

**Keywords:** ultra-processed foods, PRISm, lung function, NHANES, pre-COPD

## Abstract

**Background:**

Preserved Ratio Impaired Spirometry (PRISm) is increasingly recognized as a precursor to Chronic Obstructive Pulmonary Disease (COPD). The impact of Ultra-Processed Foods (UPFs) intake on PRISm and lung function remains underexplored, and we aimed to explore their associations.

**Methods:**

This study included 8,336 U.S. adults. Weighted logistic and linear regression models were employed for main analysis. Dose–response relationship was examined through restricted cubic spline (RCS) analysis, and subgroup analyses explored interactions with selected covariates.

**Results:**

Participants in the PRISm group were older and exhibited various adverse health characteristics. The percentage of total daily energy intake from UPFs (%Kcal) intake was associated with a non-significant increase in PRISm risk (OR 1.67, 95% CI: 0.96–2.92, *p* = 0.07). However, the highest quartile of UPFs (%Kcal) intake was significantly linked to increased PRISm risk (OR 1.36, 95% CI: 0.99–1.86, P for trend = 0.043). Furthermore, higher UPFs (%Kcal) intake negatively affected lung function, with participants in the highest quartile showing a significant reduction in forced expiratory volume in 1 s (FEV1) of −45.5 mL (95% CI: −87.6 to −3.4, P for trend = 0.045) and a decrease in forced vital capacity (FVC) of −139.4 mL (95% CI: −223.5 to −55.4, *p* < 0.001) compared to those in the lowest quartile. RCS analysis demonstrated linear relationships for both PRISm and lung function. Subgroup analysis revealed increased susceptibility primarily among individuals with occupational exposure. Additionally, sensitivity analysis indicated that a higher percentage of total daily intake from UPFs (%Grams) intake was significantly associated with an increased risk of PRISm (OR 1.86, 95% CI: 1.07–3.25, *p* = 0.03).

**Conclusion:**

Higher intake of UPFs is linked to an increased risk of PRISm and negatively affects lung function, particularly in individuals with occupational exposure.

## 1 Introduction

Ultra-processed foods (UPFs) are foods and beverages produced through a series of complex industrial processes, often with the addition of food additives to enhance taste, appearance, aroma, and shelf life. These products undergo numerous processing steps, most of which cannot be replicated at home, and the final product typically bears little resemblance to its original ingredients (1). These foods undergo multiple processing stages that alter their original composition, introducing additives and preservatives that enhance flavor, prolong shelf life, and increase convenience (2). In recent years, UPFs consumption has surged globally due to factors such as urbanization, aggressive marketing, and lifestyle changes. This trend raises significant public health concerns, as UPFs are associated with low dietary quality and linked to various adverse health outcomes (3). Numerous studies have established strong associations between high UPFs intake and the risk of chronic diseases, including cardiovascular disorders (4), obesity (5), type 2 diabetes (6), certain cancers (7), and respiratory health (8).

Preserved ratio impaired spirometry (PRISm) is a respiratory condition characterized by a normal ratio of forced expiratory volume in 1 s (FEV1) to forced vital capacity (FVC), despite a reduction in FEV1 (9). PRISm serves as an important indicator of early lung function decline and is frequently viewed as a precursor to chronic obstructive pulmonary disease (COPD) (9). Its prevalence is particularly high among adults, especially those who smoke or are exposed to environmental pollutants (10–12). Identifying PRISm and its associated risk factors is crucial, as this stage allows for timely intervention to prevent further deterioration of lung function and the progression to COPD, which can lead to symptoms such as shortness of breath and decreased physical ability, ultimately affecting quality of life and increasing healthcare demands (13).

Dietary is recognized as a critical factor in influencing the prognosis of respiratory diseases. Many studies have demonstrated that healthy dietary patterns, as measured by indices like the Dietary Inflammatory Index (DII) and the Composite Dietary Antioxidant Index (CDAI), can help reduce the risk of COPD and its mortality (14, 15). However, there is a notable lack of research exploring the relationship between dietary patterns and PRISm. Therefore, this study aims to explore the relationship between UPFs intake and PRISm among adults using data from the National Health and Nutrition Examination Survey (NHANES), providing valuable insights into how dietary influence PRISm and lung function.

## 2 Materials and methods

### 2.1 Design

**Figure 1:**
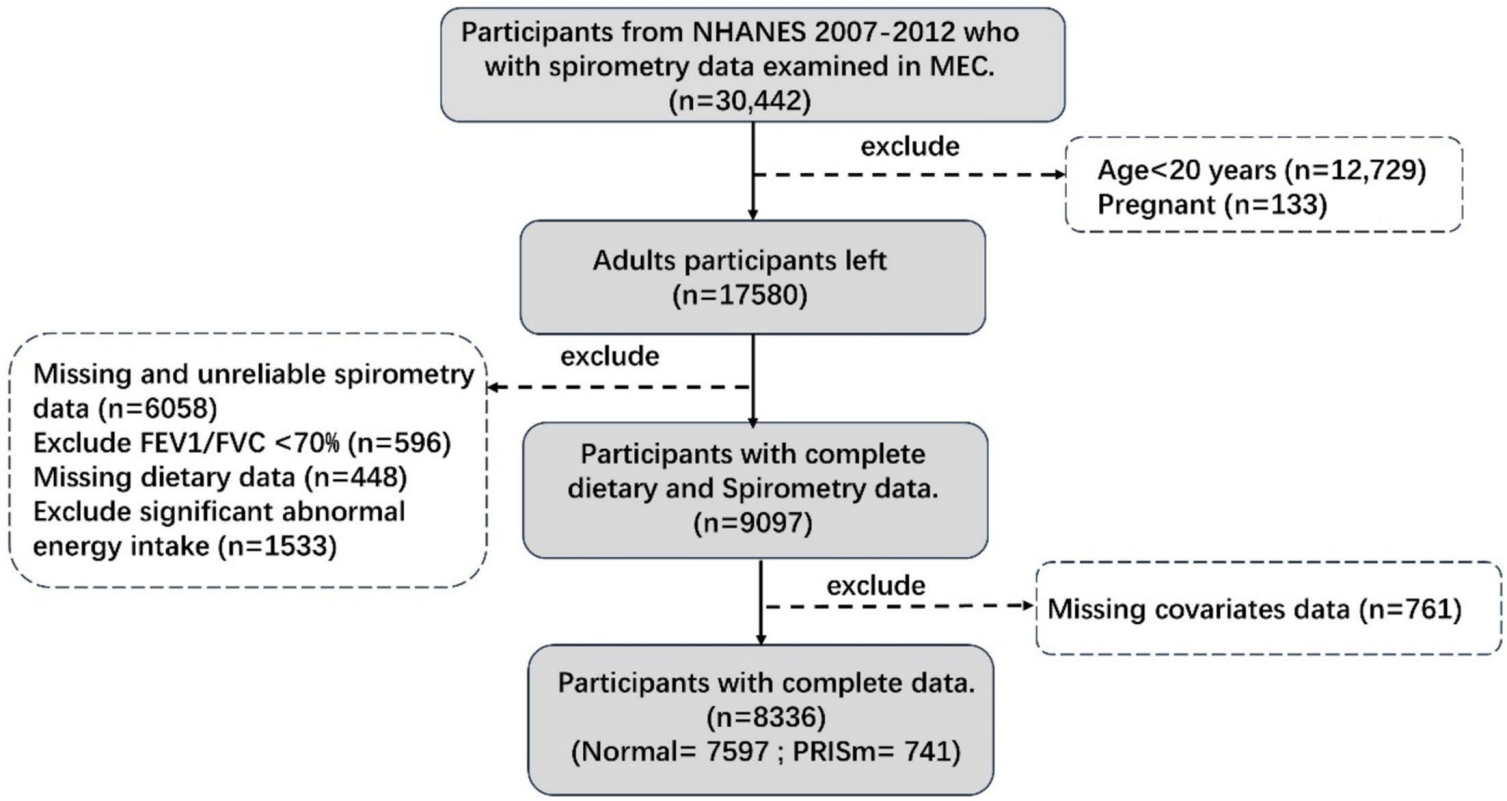
Participants flow chart. Unreliable spirometry: not exceeds or meets the American Thoracic Society data collection standards. Significant abnormal energy intake: total energy intake >5,000 kcal/d for women and > 8,000 kcal/d for men, or < 500 kcal/d.

NHANES is an ethically approved, cross-sectional study that uses scientific sampling to ensure a representative sample of the U.S. population for health examinations and surveys. This analysis utilized data from three consecutive NHANES cycles (2007–2008, 2009–2010, and 2011–2012), each of which included lung function measurements. From an initial cohort of 30,442 participants, our analyses focused on 8,336 individuals after applying specific inclusion and exclusion criteria. Participants aged ≥20 years old underwent questionnaire interviews, physical examinations, and biospecimen collection at mobile examination centers, providing comprehensive information on demographics, socio-economic status, dietary information, and spirometry results. Further methodological details are presented in Figure 1.

### 2.2 UPFs

Individual 24-h dietary recalls were collected using the USDA Multiple-Pass Method, with detailed procedures available on the NHANES website. Based on the NOVA classification, we identified the composition of individual UPFs consumption, recording intake in both calories and grams. We then calculated the percentage of total daily energy intake from UPFs (%Kcal) and the percentage of total daily gram intake from UPFs (%Gram), which served as indicators of UPFs consumption. Further methodological details can be found in our previous publication (16).

### 2.3 Measurements of spirometry and PRISm definition

Using the pulmonary function parameters from the NHANES SPX dataset, we collected FEV1 and FVC as primary indicators and calculated the FEV1/FVC ratio. PRISm was defined as FEV1/FVC ≥ 0.7 with FEV1% < 80% of the predicted value, where the predicted values were calculated based on the NHANES III equations (17, 18).

### 2.4 Covariation

Our analysis included a range of covariates known or assumed to be associated with lung function and dietary quality. These covariates were age (as a continuous variable or categorized as <40, 40–59, and ≥ 60 years), sex (female, male), race/ethnicity (non-Hispanic Black, non-Hispanic White, Mexican American, and Others), poverty-to-income ratio (PIR: low <1.3, middle 1.3–3.5, high >3.5), and BMI based on WHO classifications (low-normal, overweight, obese). Education level was categorized as college or higher, high school, and middle school or lower. Alcohol intake was classified as nondrinkers, mild–moderate drinkers (1–3 drinks/day), and heavy drinkers (≥4 drinks/day) (19), while smoking status was divided into current, former, and never smokers. Physical activity level was classified as active, moderate, inactive, or other (20). Additionally, mean daily energy intake was calculated from 2 days’ dietary data. Height was also considered in the analysis of FEV1, FVC, and FEV1/FVC.

### 2.5 Statistical methods

To accommodate the NHANES complex sampling design, sample weights (Dietary day one sample weight) were applied to all analyses. Continuous variables were presented as weighted means with standard errors (SE), while categorical variables were reported as weighted percentages (SE). Differences in continuous variables between the normal and PRISm groups were analyzed using Student’s t-test, and differences in categorical variables were assessed with the Cochran–Mantel–Haenszel Chi-square test.

Weighted logistic regression was employed to evaluate associations between UPFs (%Kcal) intake and PRISm. Model 1 was unadjusted, while Model 2 adjusted for age, sex, and ethnicity. Model 3 included further adjustments for BMI, PIR, education, physical activity, smoking, alcohol intake, occupational exposure, and average energy intake. Weighted linear regression analyses were conducted to examine associations between UPFs (%Kcal) intake and lung function, utilizing the same three models; however, Model 3 additionally adjusted for height. Restricted cubic spline (RCS) analysis was performed to explore the dose–response relationships between UPFs (%Kcal) intake and both PRISm and lung function, adjusting for all potential confounders. Subsequent subgroup and interaction analyses were also conducted. Finally, a sensitivity analysis was performed to examine the relationships between UPFs (%Gram) intake and both PRISm and lung function parameters. All statistical analyses were conducted using R version 4.4.1.

## 3 Results

### 3.1 Participant characteristics

This study included a total of 8,336 U.S. adults, among whom 741 participants were classified in the PRISm group. Participants in the PRISm group were significantly older, with a mean age of 48.2 years, compared to 43.1 years in the normal group. This group was more likely to be non-Hispanic Black individuals, had a higher BMI, lower education levels, lower PIR, inactive physical activity, higher rates of active smoking, a greater proportion of non-drinkers, as well as lower occupational exposure and energy intake. Detailed characteristics are presented in Table 1.

**Table 1:** Characteristics of the study participants among U.S adults (NHANES 2007–2012).

Variable	Total	Normal	PRISm	
	*n* = 8,336	*n* = 7,597	*n* = 741	*p* value
Age	43.4 (0.4)	43.1 (0.4)	48.2 (0.8)	< 0.001
Sex				0.5
Female	51.0 (0.0)	50.9 (0.8)	52.5 (2.4)	
Male	49.0 (0.0)	49.1 (0.8)	47.5 (2.4)	
Ethnicity				< 0.001
Non-Hispanic White	69.3 (0.0)	71.1 (2.0)	40.3 (4.8)	
Non-Hispanic Black	10.1 (0.0)	8.0 (0.8)	44.4 (4.5)	
Mexican American	8.5 (0.0)	8.9 (1.2)	2.5 (0.8)	
Others	12.1 (0.0)	12.1 (1.0)	12.9 (1.7)	
BMI (kg/m2)	28.8 (0.1)	28.7 (0.1)	31.7 (0.4)	< 0.001
BMI group				< 0.001
Low-normal	30.2 (0.0)	30.7 (1.2)	23.6 (2.4)	
Overweight	33.3 (0.0)	34.0 (1.0)	20.6 (1.7)	
Obesity	36.5 (0.0)	35.3 (0.9)	55.9 (2.6)	
Height	169.3 (0.2)	169.3 (0.2)	169.1 (0.4)	0.7
Education				< 0.001
High school	31.5 (0.0)	30.8 (1.5)	41.9 (2.7)	
Middle school or lower	4.2 (0.0)	4.1 (0.4)	4.8 (0.7)	
College or more	64.4 (0.0)	65.0 (1.6)	53.3 (2.6)	
PIR				< 0.001
High	46.6 (0.0)	47.2 (1.7)	36.1 (3.1)	
Middle	32.6 (0.0)	32.5 (1.2)	35.3 (2.5)	
Low	20.8 (0.0)	20.3 (1.1)	28.6 (2.6)	
Physical activity				< 0.001
Active	55.3 (0.0)	55.9 (1.2)	46.0 (2.8)	
Moderate	12.0 (0.0)	12.2 (0.6)	8.4 (1.3)	
Inactive	15.1 (0.0)	14.9 (0.7)	18.3 (1.7)	
Others	17.6 (0.0)	17.0 (0.8)	27.2 (2.0)	
Smoke				0.03
Never	57.7 (0.0)	58.1 (1.1)	51.1 (3.4)	
Former	22.1 (0.0)	22.0 (1.0)	24.1 (2.4)	
Now	20.2 (0.0)	19.9 (0.8)	24.9 (2.3)	
Drinker				< 0.001
Non drinker	20.5 (0.0)	20.9 (0.9)	32.6 (2.5)	
Mild–moderate	50.9 (0.0)	53.6 (1.2)	51.0 (2.5)	
Heavy	23.8 (0.0)	25.5 (0.9)	16.5 (2.6)	
Occupational exposure	51.5 (0.0)	51.7 (1.1)	48.7 (2.8)	0.2
Energy intake (kcal)	3806.5 (25.3)	3823.1 (24.7)	3535.1 (84.2)	< 0.001
UPFs(%kcal)	0.5 (0.0)	0.5 (0.0)	0.5 (0.0)	0.01
UPFs(%Gram)	0.3 (0.0)	0.3 (0.0)	0.4 (0.0)	0.002
Lung function				
FEV1, mL	3339.9 (16.3)	3403.1 (16.4)	2309.0 (29.8)	< 0.001
FVC, mL	4175.2 (17.4)	4248.9 (17.2)	2974.2 (35.4)	< 0.001
FEV1/FVC, %	80.1 (0.1)	80.2 (0.1)	77.8 (0.4)	< 0.001

Continuous variable was presented as mean (SE) and categorical variables were presented as percentages (SE). Variables between groups were compared by Student’s t-tests and chi-square tests.

### 3.2 Associations between UPFs and PRISm and lung function

Table 2 summarizes the associations between UPFs (%Kcal) intake and PRISm and lung function, as assessed through weighted logistic and linear regression analyses. After adjusting for all covariates, continuous UPFs (%Kcal) intake was associated with a non-significant increase in the risk of PRISm (OR 1.67, 95% CI: 0.96–2.92, *p* = 0.07). However, when categorized into quartiles, the highest quartile of UPFs (%Kcal) intake was significantly associated with an increased risk of PRISm compared to the lowest quartile (OR 1.36, 95% CI: 0.99–1.86, P for trend = 0.043). Additionally, higher UPFs (%Kcal) intake was negatively correlated with lung function. Specifically, participants in the highest quartile exhibited a significant reduction in FEV1 of −45.5 mL (95% CI: −87.6 to −3.4, P for trend = 0.045) and a decrease in FVC of −139.4 mL (95% CI: −223.5 to −55.4, *p* < 0.001) compared to those in the lowest quartile. The continuous analysis of UPFs (%Kcal) intake also revealed a significant reduction in FVC of −87.4 mL (95% CI: −135.7 to −39.1, P for trend <0.001). Furthermore, continuous UPFs (%Kcal) intake was associated with a modest increase in the FEV1/FVC ratio of 1.0 (95% CI: 0.3–1.7, *p* = 0.01), with significant associations noted in the highest quartile (0.5, 95% CI: 0.1–1.0, P for trend = 0.019).

**Table 2:** Associations between UPFs (%Kcal) and PRISm and lung function.

	Model 1		Model 2		Model 3	
Character	Estimates (95%CI)	*p* value	Estimates (95% CI)	*p* value	Estimates (95%CI)	*p* value
PRISm	1.92 (1.17, 3.15)	0.01	1.94 (1.19, 3.15)	0.01	1.67 (0.96, 2.92)	0.07
Q1	ref	ref	ref	ref	ref	ref
Q2	0.89 (0.63, 1.26)	0.51	0.98 (0.68, 1.41)	0.91	0.94 (0.64, 1.38)	0.75
Q3	0.98 (0.72, 1.34)	0.9	1.03 (0.75, 1.42)	0.84	1.00 (0.71, 1.42)	1
Q4	1.38 (1.05, 1.82)	0.02	1.46 (1.11, 1.93)	0.01	1.36 (0.99, 1.86)	0.05
p for trend		0.011		0.007		0.043
FEV1	277.4 (152.9, 401.9)	<0.001	−87.6(−156.3, −18.9)	0.01	−61.5(−135.7, 12.6)	0.1
Q1	ref	ref	ref	ref	ref	ref
Q2	36.7(−31.0, 104.4)	0.3	−5(−36.9, 27.0)	0.8	−10.4(−42.2, 21.4)	0.5
Q3	76.5(−4.3, 157.2)	0.1	−28.8(−66.6, 8.9)	0.1	−27.6(−66.1, 11.0)	0.2
Q4	122.8 (50.9, 194.7)	0.001	−57.2(−94.8, −19.6)	0.004	−45.5(−87.6, −3.4)	0.04
p for trend		<0.001		0.003		0.045
FVC	202.6 (59.3, 345.9)	0.01	−174.8(−251.1, −98.6)	<0.001	−139.4(−223.5, −55.4)	0.002
Q1	ref	ref	ref	ref	ref	ref
Q2	23.3(−62.3, 108.9)	0.6	−22.1(−64.8, 20.5)	0.3	−26.1(−66.7, 14.5)	0.2
Q3	61.6(−33.3, 156.4)	0.2	−55.2(−96.5, −14.0)	0.01	−48.7(−95.1, −2.3)	0.04
Q4	74.1(−10.7, 158.9)	0.1	−107.2(−149.1, −65.3)	<0.001	−87.4(−135.7, −39.1)	0.001
p for trend		0.051		<0.001		0.001
FEV1/FVC	2.7 (1.7, 3.7)	<0.001	1.1 (0.4, 1.8)	0.005	1.0 (0.3, 1.7)	0.01
Q1	ref	ref	ref	ref	ref	ref
Q2	0.4(−0.1, 0.9)	0.11	0.3(−0.2, 0.8)	0.23	0.3(−0.2, 0.8)	0.27
Q3	0.6 (0.1, 1.1)	0.01	0.3(−0.1, 0.8)	0.15	0.3(−0.2, 0.8)	0.19
Q4	1.5 (0.9, 2.0)	<0.001	0.6 (0.2, 1.0)	0.01	0.5 (0.1, 1.0)	0.02
p for trend		<0.001		0.008		0.019

Model 1: adjusted for none. Model 2: adjusted for age, sex, ethnicity. Model 3: adjusted for age, sex, ethnicity, BMI, PIR, education, physical activity, smoke, drinks, occupational exposure, and average energy intake. In lung function model, height was additionally adjusted.

Estimates for PRISm are expressed as odds ratios (OR) derived from logistic regression models, with UPFs analyzed as both a continuous variable and a categorical variable (quartiles) and, lung function estimates are presented as coefficients derived from linear regression models, with UPFs also analyzed as both a continuous variable and a categorical variable (quartiles).

Ref: reference.

### 3.3 Dose–response relationship

**Figure 2:**
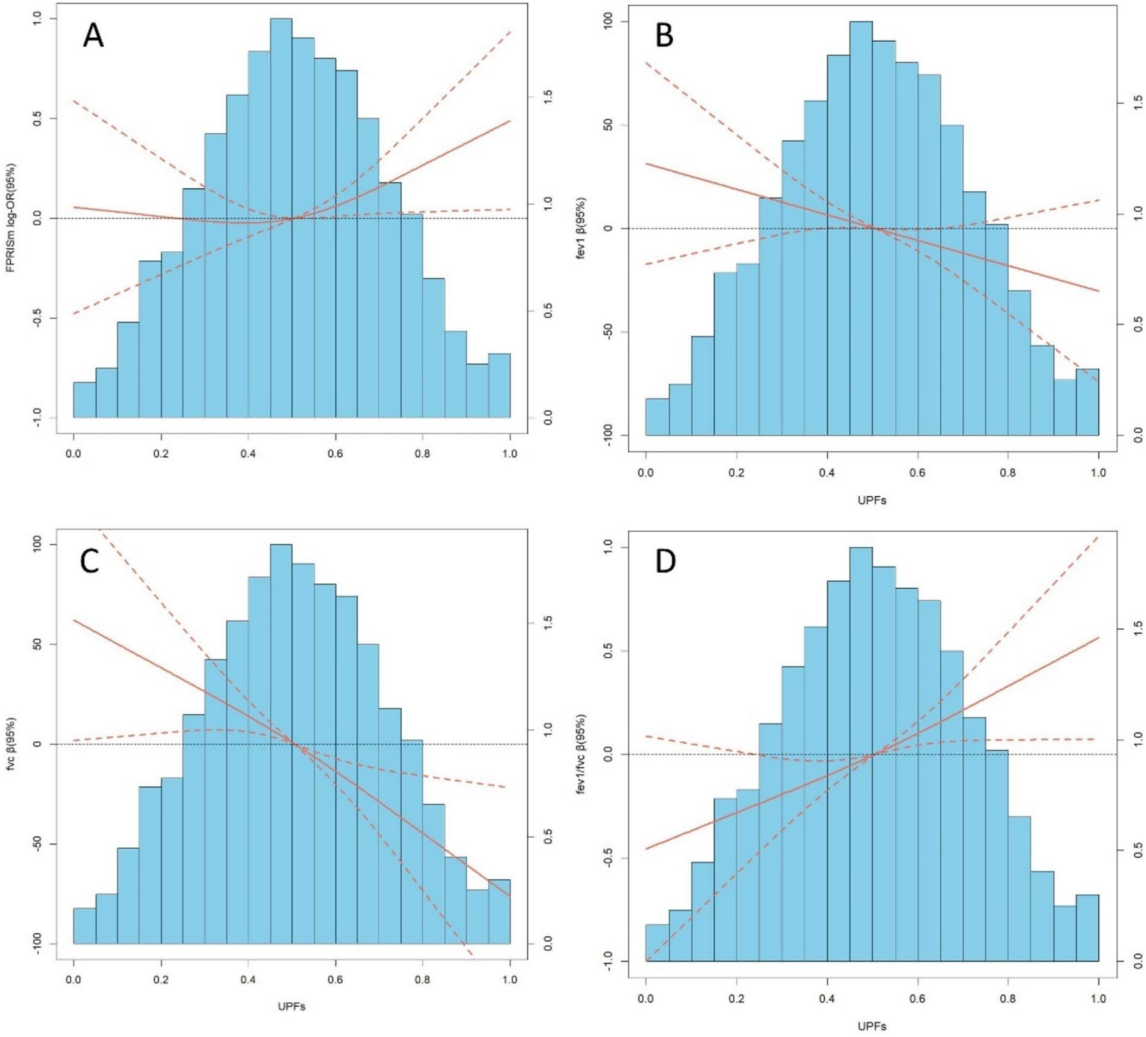
**(A–D)** depict distributions of frequency of UPFs (%Kcal) and dose–response relationship between UPFs (%Kcal) and PRISm and lung function in the sample of 8,336 U. S adults from NHANES 2007 to 2012. Red solid lines and Red dotted line represent RCS models and 95%CI, respectively. Multivariable linear regression model is used to estimate the fully adjusted estimates in PRISm and lung function and corresponding 95%CI. Models were adjusted by age, sex, ethnicity, BMI, PIR, education, physical activity, smoke, drinks, occupational exposure, and average energy intake. In lung function model, height was additionally adjusted.

RCS analysis revealed linear relationships between UPFs, PRISm, and lung function (Figure 2). A linear association for PRISm was observed (P overall = 0.067, P nonlinearity = 0.196). Significant linear associations were found for FEV1 (P overall = 0.0411, P nonlinearity = 0.9942), FVC (P overall <0.001, P nonlinearity = 0.7524), and FEV1/FVC (P overall = 0.0008, P nonlinearity = 0.7941).

### 3.4 Subgroup and interaction analysis

Stratified analysis by selected covariates revealed differential associations between UPFs (%Kcal) intake, PRISm, and lung function (Table 3). Individuals with occupational exposure demonstrated heightened susceptibility to the effects of UPFs (%Kcal) intake on PRISm, FVC, and FEV1/FVC, with significant interactions noted (P for interaction = 0.035, 0.036, and 0.023, respectively). Moreover, UPFs (%Kcal) showed a stronger impact on FEV1 among non-Hispanic Black individuals (P for interaction = 0.019). For FVC, the greatest effects were observed in males and both non-Hispanic White and Black populations, with interaction *p*-values of 0.024 and 0.039.

**Table 3:** Subgroup and interaction analyses between UPFs (%Kcal) and PRISm and lung function, stratified by selected covariates.

	PRISm		FEV1		FVC		FEV1/FVC	
Character	OR (95% CI)	p*	*β* (95% CI)	p*	*β* (95% CI)	p*	*β* (95% CI)	p*
Age		0.745		0.222		0.267		0.908
< 40	1.19 (0.56, 2.57)		−50.7 (−175.8, 74.5)		−144.4 (−267.8, −21.0)		1.3 (0.1, 2.6)	
40–59	2.46 (0.99, 6.12)		−66.1 (−194.3, 62.1)		−101.8 (−257.1, 53.4)		0.3 (−0.8, 1.4)	
≥ 60	1.91 (0.48, 7.54)		−202.1 (−395.2, −9.0)		−274.1 (−529.9, −18.4)		0.2 (−1.2, 1.7)	
Sex		0.106		0.131		0.024		0.919
Female	1.27 (0.62, 2.57)		−32.4 (−120.2, 55.3)		−57.0 (−151.1, 37.1)		0.6 (−0.7, 1.8)	
Male	2.40 (1.08, 5.33)		−124.3 (−241.9, −6.7)		−230.4 (−372.3, −88.4)		0.9 (−0.2, 2.0)	
Ethnicity		0.36		0.019		0.039		0.339
Non-Hispanic White	2.45 (0.89, 6.72)		−114.0 (−239.9, 11.9)		−190.5 (−328.7, −52.4)		0.7 (−0.3, 1.7)	
Non-Hispanic Black	1.49 (0.62, 3.60)		−153.7 (−287.0, −20.4)		−176.7 (−303.9, −49.5)		−0.2 (−1.8, 1.4)	
Mexican American	0.57 (0.04, 8.29)		106.8 (−75.1, 288.8)		24.5 (−219.3, 268.2)		2.2 (0.5, 3.8)	
Others	1.07 (0.24, 4.71)		67.8 (−60.9, 196.5)		35.0 (−145.8, 215.8)		1.0 (−0.8, 2.9)	
PIR		0.888		0.483		0.351		0.955
High	2.08 (0.61, 7.05)		−117.8 (−264.8, 29.2)		−188.6 (−358.4, −18.8)		0.6 (−0.5, 1.8)	
Middle	2.15 (0.88, 5.26)		−67.7 (−192.5, 57.0)		−114.6 (−235.6, 6.4)		0.5 (−0.8, 1.8)	
Low	1.40 (0.54, 3.61)		−26.3 (−126.7, 74.1)		−109.0 (−222.1, 4.1)		1.3 (−0.3, 3.0)	
BMI group		0.245		0.967		0.284		0.011
Low-normal	0.86 (0.24, 3.09)		−120.6 (−277.3, 36.2)		−259.1 (−390.9, −127.4)		2.0 (0.1, 3.9)	
Overweight	2.52 (0.76, 8.41)		−62.5 (−190.3, 65.4)		−100.1 (−247.3, 47.2)		0.1 (−1.2, 1.3)	
Obesity	2.38 (1.24, 4.55)		−86.7 (−237.1, 63.8)		−109.0 (−279.5, 61.5)		0.1 (−1.0, 1.2)	
Smoke		0.544		0.882		0.876		0.821
Never	1.45 (0.76, 2.76)		−93.6 (−216.9, 29.7)		−150.7 (−280.3, −21.2)		0.7 (−0.4, 1.8)	
Former	1.43 (0.35,5.81)		−95.5 (−258.2, 67.3)		−176.3 (−362.2, 9.6)		0.6 (−0.9, 2.2)	
Now	2.28 (0.73, 7.14)		−7.8 (−162.3, 146.7)		−76.9 (−256.7, 102.9)		1.1 (−0.7, 3.0)	
Physical activity		0.474		0.679		0.46		0.462
Active	1.87 (0.81, 4.32)		−111.8 (−221.6, −1.9)		−182.7 (−292.4, −73.1)		0.5 (−0.8, 1.7)	
Moderate	0.98 (0.18, 5.45)		−7.8 (−151.4, 135.8)		−91.8 (−280.7, 97.1)		1.7 (−0.2, 3.7)	
Inactive	4.30 (0.96, 19.23)		−94.6 (−308.4, 119.2)		−195.6 (−463.4, 72.1)		1.6 (0.1, 3.2)	
Occupational exposure		0.035		0.163		0.036		0.023
Yes	2.63 (1.13, 6.13)		−112.6 (−208.7, −16.5)		−226.3 (−330.2, −122.5)		1.3 (0.4, 2.2)	
No	1.27 (0.62, 2.62)		−60.6 (−185.4, 64.2)		−67.5 (−217.6, 82.6)		−0.0 (−1.2, 1.1)	
Drinker		0.234		0.849		0.532		0.59
Nondrinker	0.97 (0.45, 2.08)		4.9 (−148.1, 157.9)		−16.5 (−185.1, 152.1)		0.7 (−0.8, 2.3)	
Mild–moderate	2.07 (0.99, 4.32)		−111.7 (−235.8, 12.4)		−203.8 (−340.7, −66.9)		1.0 (−0.2, 2.1)	
Heavy	3.93 (1.08, 14.23)		−85.9 (−221.7, 49.8)		−135.1 (−294.2, 24.1)		0.4 (−1.3, 2.0)	

Models were adjusted by age, sex, ethnicity, BMI, PIR, education, physical activity, smoke, drinks, occupational exposure, and average energy intake. In lung function model, height was additionally adjusted. The subgroup variable was not included in same subgroup analysis.

p*p*: p for interaction.

Estimates for PRISm are expressed as odds ratios (OR) derived from logistic regression models, with UPFs analyzed as both a continuous variable and, lung function estimates are presented as coefficients derived from linear regression models, with UPFs also analyzed as continuous variable.

### 3.5 Sensitivity analysis

To account for the fact that some additives in UPFs may not be measured in calories, we also analyzed the relationship between UPFs (%Grams) intake and PRISm and lung function (Table S1). Our findings indicated that the results remained consistent, showing that UPFs (%Grams) intake were significantly associated with an increased risk of PRISm (OR 1.86, 95% CI: 1.07–3.25, *p* = 0.03).

## 4 Discussion

This study identified significant associations between UPFs intake and both PRISm and lung function. Participants with higher UPFs (%Kcal) consumption exhibited an increased risk of PRISm compared to those with lower intake levels. Additionally, a marked decline in critical lung function indicators, specifically FEV1 and FVC, was observed among individuals consuming greater amounts of UPFs (%Kcal). Stratified analyses and interaction tests further revealed a stronger association between UPFs and PRISm among individuals exposed to occupational environments. Notably, UPFs intake measured in grams was more strongly correlated with the increased risk of PRISm.

PRISm is an unstable classification and may serve as a precursor to COPD, preventing its progression is essential (13, 21, 22). Just as COPD requires nutritional support and management, the role of dietary patterns in PRISm is increasingly recognized (23). However, evidence regarding the impact of UPFs is lacking. Previous studies have primarily focused on the relationship between UPFs consumption and COPD risk, for instance, a prospective cohort study from the UK Biobank found a correlation between UPFs intake and increased COPD risk (24). Moreover, UPFs represent a typical western dietary pattern characterized by high consumption of processed meats, refined grains, desserts, and sugary foods, and evidence showed the western dietary pattern are associated with a higher risk of COPD (25–27). Notably, specific components related to UPFs, such as processed red meat, have been linked to a 26% increase in COPD risk (28). Recent investigations have begun to explore the association between the DII and CDAI with PRISm, highlighting the potential significance of anti-inflammatory and antioxidant diets in this context (29). Our study further enhances the understanding of dietary patterns’ effects on PRISm and extends the linkage between high UPFs consumption and respiratory health. The main results and sensitivity analyses indicated potential adverse impacts of UPFs on PRISm and lung function, regardless of whether intake was measured in grams or energy, underscoring concerns about the elevated risk of PRISm associated with high UPFs consumption. Specifically, UPFs were found to be negatively associated with both FEV1 and FVC, suggesting that managing the degree of food processing may be critical in the prevention and management of PRISm. Additionally, our findings indicate that individuals with occupational exposure may be at an increased risk of PRISm, likely due to from prolonged exposure, thereby calling for sufficient nutrient support to sustain immune defense responses (30). Furthermore, we observed variations in FEV1 and FVC related to race and sex, and the underlying reasons for these differences warrant further investigation.

Mechanistically, the adverse effects of UPFs on lung function are well-documented. Classified according to the NOVA classification, UPFs often lose a significant amount of nutrients during industrial processing and are characterized by high levels of added sugars and food additives, leading to their reputation for poor nutritional quality (31). Numerous studies have demonstrated that deficiencies in nutrients, including fruits, vegetables, and vitamins, negatively affect respiratory health (32, 33). The absence of these nutrients may compromise immune defense responses, contributing to inflammation and oxidative imbalance (34–36). Additionally, the high-fat, high-sugar, and low-quality protein characteristics of UPFs can adversely affect metabolic processes (37). Previous research has established the metabolic indicators such as the ratio of non-high-density lipoprotein cholesterol to high-density lipoprotein cholesterol, Triglyceride-Glucose Index (TyG), low-density lipoprotein cholesterol, and albumin can lead to chronic airway inflammation and sustained respiratory tissue damage (38–41). Moreover, the high sugar and fat content of UPFs, combined with exposure to food additives and microplastics from packaging, may disrupt gut microbiota. This dysbiosis can subsequently influence lung health through the gut-lung axis, thereby affecting pulmonary immune homeostasis—a concern that is increasingly being acknowledged in the literature (42–44).

Our research fills a critical gap by addressing the limitations of previous studies that focused on individual nutrients or food groups in relation to COPD. We have expanded the knowledge about the complex relationships between food processing, dietary combinations, and the risk of PRISm. However, this study still has limitations. As with all cross-sectional studies, it cannot establish causal relationships. Although we adjusted for multiple confounding factors and conducted sensitivity analyses, residual confounding cannot be entirely ruled out. Dietary assessments conducted through questionnaires may introduce measurement error, and since NHANES did not classify diets according to the NOVA classification, there may be bias in dietary categorization. Furthermore, our analysis relied on 24-h dietary recalls, which may not accurately reflect changes in dietary habits over time. Therefore, additional clinical trials or intervention studies are necessary to further explore and confirm these findings.

In conclusion, our study provides evidence that high UPFs consumption is associated with increased risks of PRISm, FEV1, and FVC decline. This research offers novel insights into the potential for dietary interventions aimed at preventing PRISm and mitigating its progression to COPD. However, the conclusion should be interpreted with caution due to the limitations of cross-sectional design. More prospective studies and mechanistic investigations are warranted to explore these relationships further.

## Data Availability

Publicly available datasets were analyzed in this study. This data can be found here: data from NHANES collection was sponsored by the CDC, and are publicly available (https://wwwn.cdc.gov/nchs/nhanes/Default.aspx).
