# Supplementary material for: Associations between ultra-processed foods intake and preserved ratio impaired spirometry in U.S. adults
Source: Front Nutr. 2025 Jan 31;12:1523736. doi: 10.3389/fnut.2025.1523736 (PMC11825343; doi:10.3389/fnut.2025.1523736)
Supplement: Supplementary file 1 [file Table_1.DOCX]

**Online Supplementary Material**

**Table S1.** Associations between UPFs(%Grams) and PRISm and lung function.

|  | **Model 1** |  | **Model 2** |  | **Model 3** |  |
| --- | --- | --- | --- | --- | --- | --- |
| **character** | **Estimates (95% CI)** | **P value** | **Estimates (95% CI)** | **P value** | **Estimates (95% CI)** | **P value** |
| **PRISm** | 2.25(1.39,3.64) | 0.001 | 2.32(1.41, 3.81) | 0.001 | 1.86(1.07, 3.25) | 0.03 |
| Q1 | ref | ref | ref | ref | ref | ref |
| Q2 | 1.19(0.89,1.57) | 0.23 | 1.21(0.90, 1.62) | 0.21 | 1.20(0.86, 1.68) | 0.28 |
| Q3 | 1.17(0.87,1.57) | 0.29 | 1.20(0.87, 1.65) | 0.27 | 1.21(0.85, 1.74) | 0.28 |
| Q4 | 1.68(1.26,2.23) | <0.001 | 1.64(1.22, 2.20) | 0.002 | 1.49(1.08, 2.07) | 0.02 |
| **p for trend** |  | <0.001 |  | 0.002 |  | 0.023 |
| **FEV1** | 308.3 (179.5, 437.2) | <.001 | -160.6 (-237.6, -83.6) | <0.001 | -114.5 (-197.4, -31.7) | 0.01 |
| Q1 | ref | ref | ref | ref | ref | ref |
| Q2 | 84.1 (2.0, 166.1) | 0.04 | 7.1 (-23.7, 37.9) | 0.64 | 11.9 (-21.1, 45.0) | 0.46 |
| Q3 | 136.5 (52.1, 220.9) | 0.002 | -52.0 (-95.3, -8.7) | 0.02 | -46.2 (-95.8, 3.4) | 0.07 |
| Q4 | 144.9 (64.5, 225.4) | <0.001 | -79.2 (-119.8, -38.6) | <0.001 | -57.3 (-101.1, -13.6) | 0.01 |
| **p for trend** |  | <0.001 |  | <.001 |  | 0.003 |
| **FVC** | 266.1 (110.4, 421.9) | 0.001 | -229.5 (-318.6, -140.5) | <.001 | -174.3 (-268.2, -80.4) | <0.001 |
| Q1 | ref | ref | ref | ref | ref | ref |
| Q2 | 88.6 (-8.3, 185.6) | 0.07 | -0.3 (-42.0, 41.4) | 0.99 | 6.3 (-40.9, 53.5) | 0.78 |
| Q3 | 134.4 (35.5, 233.3) | 0.01 | -80.1 (-130.1, -30.0) | 0.002 | -76.5 (-135.6, -17.5) | 0.01 |
| Q4 | 128.2 (27.3, 229.1) | 0.01 | -111.1 (-159.0, -63.1) | <.001 | -83.8 (-136.0, -31.6) | 0.003 |
| **p for trend** |  | 0.013 |  | <.001 |  | <0.001 |
| **FEV1/FVC** | 2.3 (1.4, 3.1) | <.001 | 0.4 (-0.2, 1.0) | 0.16 | 0.5 (-0.2, 1.2) | 0.16 |
| Q1 | ref | ref | ref | ref | ref | ref |
| Q2 | 0.3 (-0.1, 0.8) | 0.14 | 0.2 (-0.2, 0.6) | 0.35 | 0.2 (-0.3, 0.6) | 0.42 |
| Q3 | 0.7 (0.1, 1.3) | 0.02 | 0.3 (-0.2, 0.8) | 0.24 | 0.3 (-0.2, 0.8) | 0.17 |
| Q4 | 1.0 (0.6, 1.5) | <.001 | 0.2 (-0.2, 0.6) | 0.27 | 0.2 (-0.2, 0.6) | 0.3 |
| **p for trend** |  | <0.001 |  | 0.331 |  | 0.323 |

Model 1: adjusted for none. Model 2: adjusted for age, sex, ethnicity. Model 3: adjusted for age, sex, ethnicity, BMI, PIR, education, physical activity, smoke, drinks, occupational exposure, and average energy intake. In lung function model, height was additionally adjusted.

Ref: reference.

Estimates for PRISm are expressed as odds ratios (OR) derived from logistic regression models, with UPFs analyzed as both a continuous variable and a categorical variable (quartiles). And, lung function estimates are presented as coefficients derived from linear regression models, with UPFs also analyzed as both a continuous variable and a categorical variable (quartiles).
